# ATP/P2X7 receptor/NLRP3 pathway facilitates renal tubular epithelial-myofibroblast transdifferentiation and interstitial fibrosis in rats with unilateral ureteral obstruction

**DOI:** 10.3389/fphar.2025.1598151

**Published:** 2025-05-30

**Authors:** Hui Tan, Feiyan Li, Yanchao Cui, Ziwen Li, Shiqiang Yan, Quanfeng Deng

**Affiliations:** ^1^ Department of Rehabilitation Medicine, The First Affiliated Hospital of Xi’an Jiaotong University, Xi’an, Shaanxi, China; ^2^ School of Clinical Medicine, Chengdu Medical College, Chengdu, Sichuan, China; ^3^ Department of Experiment Teaching Center of Clinical Medicine, The First Affiliated Hospital of Chengdu Medical College, Chengdu, Sichuan, China; ^4^ College of Chemistry & Pharmacy, Northwest A&F University, Yangling, Shaanxi, China; ^5^ Shenzhen Institute of Otolaryngology & Key Laboratory of Otolaryngology, Longgang Otolaryngology Hospital, Shenzhen, Guangdong, China

**Keywords:** P2X7 receptor, NLRP3, renal interstitial fibrosis, ATP, tubular epithelial-myofibroblast transdifferentiation

## Abstract

**Background:**

P2X7 receptor (P2X7R) is reported involved in renal fibrosis and the activation of NOD-like receptor protein 3 (NLRP3) inflammasome. This study aimed to investigate the role of the P2X7R and NLRP3 in renal tubular epithelial-myofibroblast transdifferentiation (TEMT) and interstitial fibrosis using a rat unilateral ureteral obstruction (UUO) model.

**Methods:**

Sprague‒Dawley rats were randomly divided into the following three groups: sham, UUO, and UUO + Brilliant Blue G (BBG). BBG (50 mg/kg/d)—an antagonist of the P2X7R—was injected intraperitoneally in UUO-treated rats. The adenosine 5′-triphosphate (ATP) concentration in kidney tissue was measured. Hematoxylin and eosin staining and Masson’s trichrome staining were used to evaluate the renal injury and the deposition of the extracellular matrix. Collagen-I, collagen-III, α-smooth muscle actin (α-SMA), P2X7R and NLRP3 expression levels were measured via immunohistochemical staining. Furthermore, the mRNA levels of α-SMA, P2X7R and NLRP3 were investigated via a reverse transcription-quantitative polymerase chain reaction.

**Results:**

Significant histopathological damage, which involved tubular dilatation, interstitial inflammation, and collagen accumulation, was observed in UUO rats and was notably alleviated via BBG administration. In the UUO group, ATP concentration increased considerably in kidney tissues; however, this concentration did not decrease following BBG treatment. Collagen-Ⅰ and -III expression levels were upregulated in UUO rats and attenuated through the administration of BBG. Furthermore, BBG administration ameliorated the accumulation of myofibroblast. P2X7R and NLRP3 protein and mRNA expressions increased notably in obstructed kidneys, whereas the protein and mRNA expression of NLRP3 appeared to reduce significantly in the BBG group. However, the mRNA level of P2X7R did not change in response to BBG treatment.

**Conclusion:**

The ATP/P2X7R/NLRP3 pathway is involved in renal TEMT and interstitial fibrosis. P2X7R antagonists attenuate renal interstitial fibrosis and may potentially be used as effective therapeutic agents.

## 1 Introduction

Chronic kidney disease (CKD) is a chronic renal dysfunction characterized by nephron loss, inflammation, myofibroblast activation, and extracellular matrix (ECM) deposition. A large number of CKDs show renal interstitial fibrosis when progressing to end-stage renal disease (ESRD) (Yuan et al., 2022).

P2X7 receptor (P2X7R) is a ligand-gated ion channel that enables the controlled influx of Na+ and Ca^2^+ and efflux of K^+^,thereby controlling ion concentrations and membrane potential polarization. P2X7R is present in nearly all tissues and organs. P2X7R stimulation is known to be responsible for the activation of myriad cellular functions, including cell division, energy metabolism, inflammation and immunity (Adinolfi et al., 2018; Kopp et al., 2019). Specific receptor functions depend on the cell and the surrounding microenvironment, especially the level of extracellular adenosine 5′-triphosphate (ATP) (Drill et al., 2021). The release of ATP—an extracellular signaling molecule—is induced by cell damage and acts as a danger signal, representing a defense mechanism in the initial inflammatory phase. P2X7R is an ATP-sensitive receptor and it responds to increased ATP levels at inflammation sites and triggers a series of cellular responses (Gentile et al., 2015). Physiologically, P2X7R is predominantly present at low levels in the kidney, in the vasculature, and microvasculature in particular. P2X7R expression is reported to be significantly upregulated in models of glomerulonephritis, acute renal ischemia‒reperfusion injury, type 1 diabetes, and unilateral ureteral obstruction (UUO) and glomerulonephritis (Menzies et al., 2017). P2X7R antagonism/deletion remarkably attenuated salt-sensitive hypertension, improved creatinine clearance, and reduced albuminuria, interstitial fibrosis, and the infiltration of inflammatory cells in Dahl salt-sensitive rats (Ji et al., 2012a; Ji et al., 2012b). P2X7R was reported involved in the mediation of tubulointerstitial injury and successive fibrosis in P2X7R knockout mice in a UUO model, which indicated its role in the mechanisms underlying inflammation and fibrosis (Goncalves et al., 2006).

The NOD-like receptor protein 3 (NLRP3) inflammasome has been verified in multiple animal models of kidney diseases, including UUO, hypertensive kidney injury, diabetic kidney disease, glomerulonephritis, and 5/6 nephrectomy (Yuan et al., 2022)^.^ This inflammasome can be activated by K+ efflux, Ca^2^+ influx, oxidized mitochondrial DNA, and lysosomal damage; it comprises the NLRP3 scaffold, the adaptor protein apoptosis speck-like protein (ASC), and the effector protein procaspase-1. As a K+ efflux channel, P2X7R is known to be involved in the activation of this type of NLRP3 inflammasome (Anton-Pampols et al., 2022). In recent years, P2X7R-induced NLRP3 activation has garnered immense attention in the field of kidney disease. Patients undergoing hemodialysis showed higher mRNA levels of P2X7R and NLRP3 in peripheral blood mononuclear cells than the healthy subjects (Granata et al., 2015). According to an *in vitro* study, ATP/P2X7R/NLRP3 pathway blockade may protect renal tubular epithelial cells from ischemia-reperfusion injury (Qian et al., 2021). Furthermore, studies have confirmed that the P2X7R/NLRP3 pathway is involved in renal functional and structural alterations and inflammation in animal models of metabolic renal injury and adriamycin nephropathy (Solini et al., 2013; Zhu et al., 2022).

By the abovementioned evidence, the ATP/P2X7R/NLRP3 pathway was hypothesized to contribute to renal interstitial fibrosis in the present study. Brilliant Blue G (BBG) is a highly selective P2X7R antagonist derived from a commonly used synthetic food dye. As a food additive, BBG is non-toxic and has been approved by the FDA (Drill et al., 2021). Herein, BBG was used to antagonize P2X7R and the role of the ATP/P2X7R/NLRP3 pathway in renal tubular epithelial-myofibroblast transdifferentiation (TEMT) and interstitial fibrosis was investigated.

## 2 Materials and methods

### 2.1 Animals and surgical protocol

Specific-pathogen-free male Sprague‒Dawley rats (weight, 200 g ± 20 g; age, 4–6 weeks). The rats were purchased from Chengdu Dossy Experimental Animals Co. Ltd. (Chengdu, Sichuan, China) and maintained in the animal facilities of Chengdu Medical College with *ad libitum* access to food and water. Strict adherence to the Regulations of Experimental Animal Administration issued by the State Committee of Science and Technology of the People’s Republic of China was ensured when handling all rats involved in this study. Approval to perform this study was obtained from the Animal Ethics Committee for Experimental Research of Chengdu Medical College (approval no: 20220106-001).

The rats were randomly categorized into the following three groups (n = 6 each): sham group, UUO group (UUO), and UUO plus BBG treatment group (UUO + BBG). UUO was performed as described previously (Xie et al., 2010) under intraperitoneal (IP) 2% pentobarbital anesthesia (0.2 mL/100 g body weight). The left ureter of anesthetized rats was ligated at two points and cut between the ligatures. The sham rats underwent left ureter exposure but not ligation. In the UUO + BBG group, BBG (Sigma‒Aldrich, Saint Louis, United States, Cat, B0770) was injected IP (dose, 50 mg/kg body weight) (Bautista-Perez et al., 2020) within 1 h of surgery and subsequently once per day, whereas the UUO and sham groups received IP doses of an equal amount of sterile saline. The rats were euthanized under pentobarbital anesthesia on postoperative day 7. Trunk blood was collected to evaluate the serum concentrations of creatinine and blood urea nitrogen (BUN). The left kidneys were harvested for further study. Partial kidney tissues were fixed in 10% formalin for histopathological and immunohistochemical (IHC) analyses. A portion of the kidney tissue was immediately used to measure ATP concentration. The remaining kidney tissues were stored immediately at −80°C for total RNA extraction.

### 2.2 Evaluation of renal function

Serum creatinine and BUN concentration were measured using a commercial enzyme-linked immunosorbent assay (ELISA) kit according to the manufacturer’s instructions (Mlbio, Shanghai, China, Cat, YY718309, YY798209). Creatinine and BUN concentrations were determined by measuring the optical density (OD) at 450 nm using a microtiter plate reader according to the manufacturer’s instructions. A standard curve was used to calculate the concentrations of creatinine and BUN in the serum samples.

### 2.3 Kidney histopathology

Kidney tissues were fixed, dehydrated in a graded ethanol series, embedded in paraffin, sectioned into 5-µm thick slices, and stained with hematoxylin and eosin (H&E) and Masson’s trichrome for histopathological analysis according to the standard protocols of the modified stain kits (Solarbio, Beijing, China, Cat G1121, G1340). The morphological structure of kidney tissues was observed in a blinded manner under a light microscope. ECM deposition was determined via Masson’s trichrome staining. For each section, 10 random nonoverlapping fields at 400× magnification were digitized and scanned using Image-Pro Plus 6.0 software in a blinded manner. The percentage of the blue collagen area in each examined section was determined.

### 2.4 Kidney tissue ATP concentration measurement

The concentration of ATP in kidney tissue was measured using an ATP detection kit according to the manufacturer’s instructions (Solarbio, Beijing, China, BC0305). The procedure was performed on ice, furthermore, OD was measured at 340 nm 10 s, 3 min and 10 s after the kidney tissue homogenate reacted with the detection reagent. The standard solution was consequently prepared and the ATP concentration was determined by calculating the ΔOD and comparing it with the standard solution.

### 2.5 IHC staining

Paraffin-embedded sections (5 µm) were deparaffinized and rehydrated before being treated with 3% H2O2 for 10 min. Heat-mediated antigen retrieval was performed using a microwave depending on the specific antibody. The sections were subsequently treated with 10% normal goat serum in phosphate buffer solution for 15 min to block nonspecific binding. Primary antibodies against rat α-smooth muscle actin (α-SMA) (1:1,000, Abcam, Cat, ab124964), P2X7R (0.1 μg/mL, Abcam, Cat, ab109054), NLRP3 (1:100, Affinity, Cat, DF7438), collagen-Ⅰ (1:500, Abcam, Cat, ab270993) and collagen-III (1:100, Abcam, Cat, ab7778) were added and incubated overnight at 4°C in a humidified chamber for approximately 16 h, followed by a horseradish peroxidase-conjugated goat anti-rabbit IgG (H + L) secondary antibody (1:500, Biodragon, Cat, BF03008X) incubation for 40 min at 37°C. The tissue sections were then incubated with diaminobenzidine (DAB) as a substrate for signal development. Regarding the negative controls, we replaced the primary antibodies with antibody dilutions. The sections were consequently counterstained with hematoxylin. Digital images were captured in 3 randomly selected fields for each section using an Olympus BX63 Microscope (Olympus, Tokyo, Japan), and then the integrated optical density (IOD) value was analyzed using Image-Pro Plus 6.0 software.

### 2.6 Reverse transcription quantitative polymerase chain reaction

We extracted total RNA from kidney tissue using All Pure RNA Extracting Kits (CWBIO, Beijing, China, Cat, CW0581S) and measured the RNA concentration with an ultraviolet spectrophotometer. In total, 1 µg of RNA was reverse-transcribed using a HiScript III RT Supermix for qPCR Kit (Vazyme, Nanjing, China, Cat, R323). Reverse transcription-quantitative polymerase chain reaction (RT-qPCR) was performed using the SYBR green method. Primers for α-SMA, P2X7R, NLRP3, and the internal control glyceraldehyde-3-phosphate dehydrogenase (GAPDH) (synthesized by Servicebio, Wuhan, China) are listed in Table 1. PCR amplifications were performed according to the instructions of Taq Pro Universal SYBR qPCR Master Mix (Vazyme, Nanjing, China, Cat, Q712) on a Bio-Rad CFX Connect Real-Time PCR Detection System. The relative levels of target gene mRNA expression were calculated by the cycle threshold (CT) and normalized by GAPDH by using the 2^−ΔΔCT^ comparative method.

**TABLE 1:** The sequences of the forward and reverse primers for PCR.

Gene name	Primers
α-SMA	Forward 5′ TCCTTCTATAACGAGCTTCGC 3′ Reverse 5′ TCTCCAGAGTCCAGCACAATAC 3′
P2X7R	Forward 5′ CCCTGTCCTATTTCGGTTTGG 3′ Reverse 5′ GTCTCGGGACCTCTTGACCTTT 3′
NLRP3	Forward 5′ CCTCAACAGACGCTACACCC 3′ Reverse 5′ CCACATCTTAGTCCTGCCAAT 3′
GAPDH	Forward 5′ CTGGAGAAACCTGCCAAGTATG 3′ Reverse 5′ GGTGGAAGAATGGGAGTTGCT 3′

Abbreviations: α-SMA, α-smooth muscle actin; P2X7R, P2X7 receptor; NLRP3, NOD-like receptor protein 3; GAPDH, glyceraldehyde-3-phosphate dehydrogenase.

### 2.7 Statistical analyses

Statistical analyses were performed using SPSS software 26.0. All data are shown as the mean ± standard deviation. Levene’s test was used to examine the homogeneity of variance. Intergroup comparisons were performed using one-way analysis of variance (ANOVA) followed by *post hoc* tests with the Bonferroni test or Dunnett’s T3 test based on whether the variance was equal. P < 0.05 indicated statistical significance.

## 3 Results

### 3.1 Kidney histopathological changes

**FIGURE 1:**
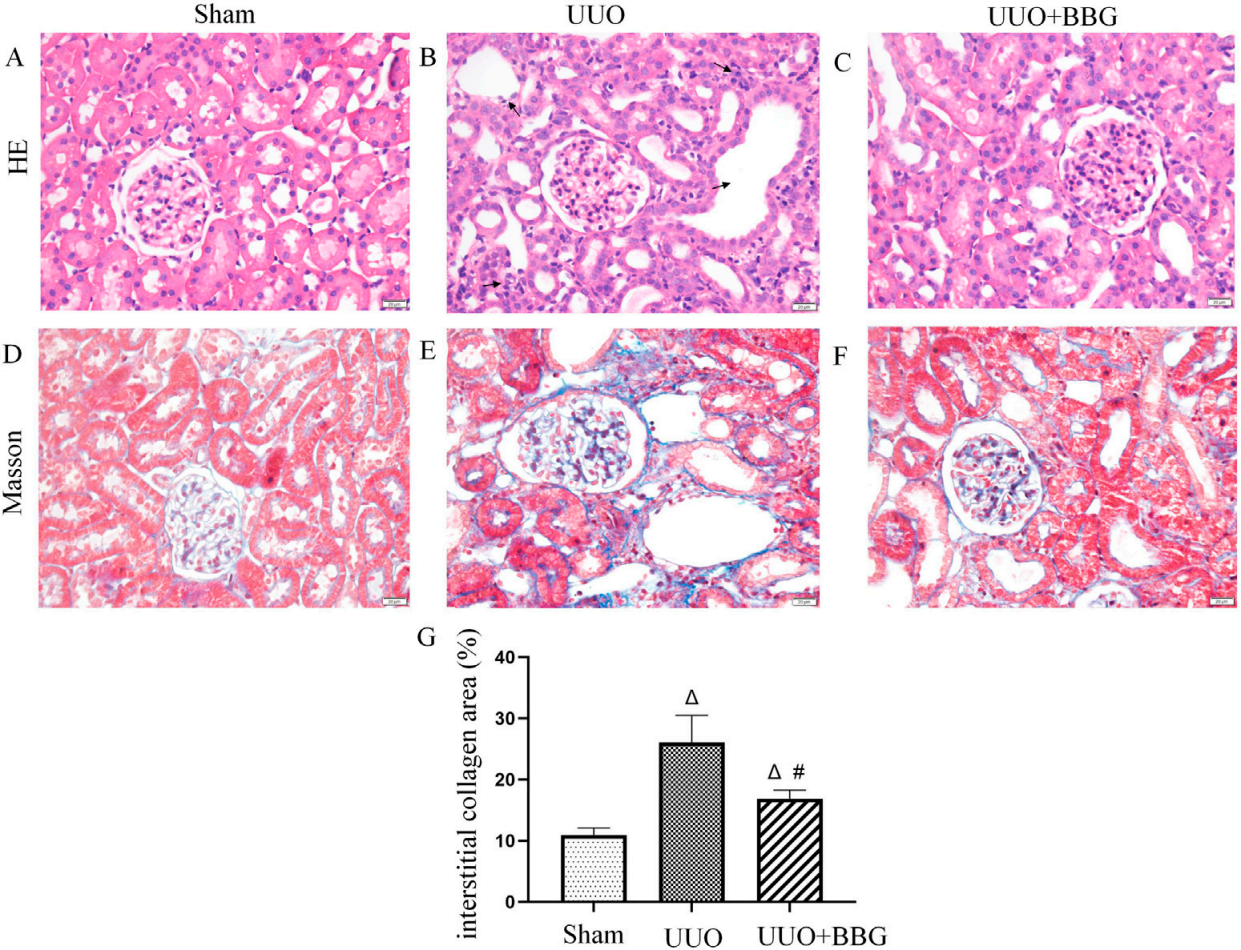
Histopathological changes in kidneys. Hematoxylin and eosin staining show normal glomeruli and tubules in the sham group **(A)**, tubular dilatation and atrophy and interstitial inflammation are visible (→indicate) in the unilateral ureteral obstruction (UUO) group **(B)** and alleviated renal histopathological injury is evident after Brilliant Blue G (BBG) treatment **(C)**. Obstructed kidneys show significant collagen accumulation **(E)**, which is considered minimal in normal kidneys **(D)**. BBG treatment inhibits the accumulation of collagen in the kidneys of UUO rats **(F)**. Statistical analyses of area showing interstitial collagen deposition showed intergroup differences **(G)**. Scale bar = 20 μm. Magnification ×400. ∆, P < 0.05 vs. sham group; #, P < 0.05 vs. UUO group.

Histopathological features were observed in H&E− and Masson trichrome-stained sections (Figure 1). UUO animals exhibited prominent tubular dilatation and atrophy, mononuclear cell infiltration, interstitial inflammation (Figure 1B), collagen accumulation, and fibrosis (Figure 1E). No histological abnormalities were observed in sham-operated kidneys (Figures 1A, D). After blocking P2X7R with BBG, tubulointerstitial injury and collagen deposition appeared to be partially alleviated (Figures 1C, F). Figure 1G shows the intergroup differences in the collagen area percentages.

### 3.2 Renal ATP concentrations

**FIGURE 2:**
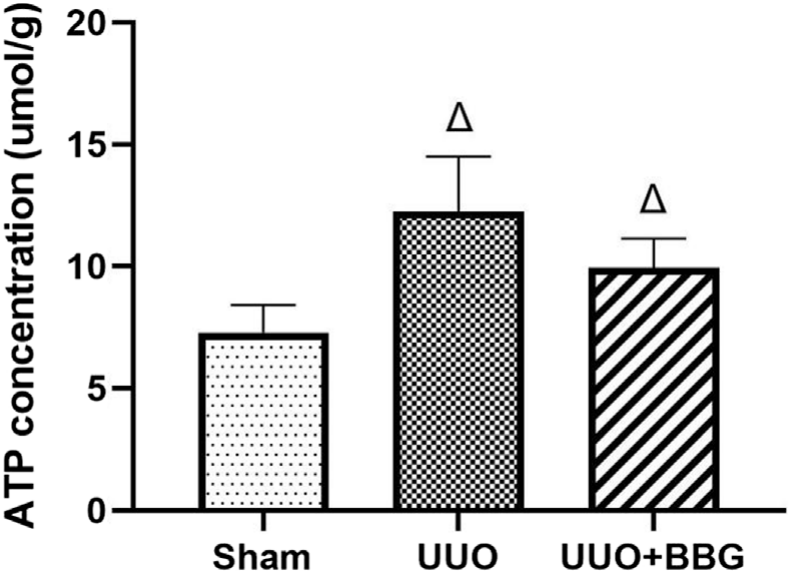
Renal adenosine 5′-triphosphate (ATP) concentrations. A significant increase in ATP is evident in the UUO group. BBG treatment did not change ATP levels in obstructed kidneys. ∆, P < 0.05 vs. sham group.

Increased ATP concentration can activate P2X7R and, consequently, exert its effects. To elucidate the relationship between ATP and P2X7R, ATP concentrations in the kidney tissue of rats were measured. Figure 2 shows that there was a significant increase in ATP in the UUO group compared with the sham group (P < 0.05). However, no significant alterations were noted in the ATP concentrations in the obstructed kidney tissue following BBG treatment, even though a decreasing trend was observed.

### 3.3 BBG alleviated renal TEMT and interstitial fibrosis

**FIGURE 3:**
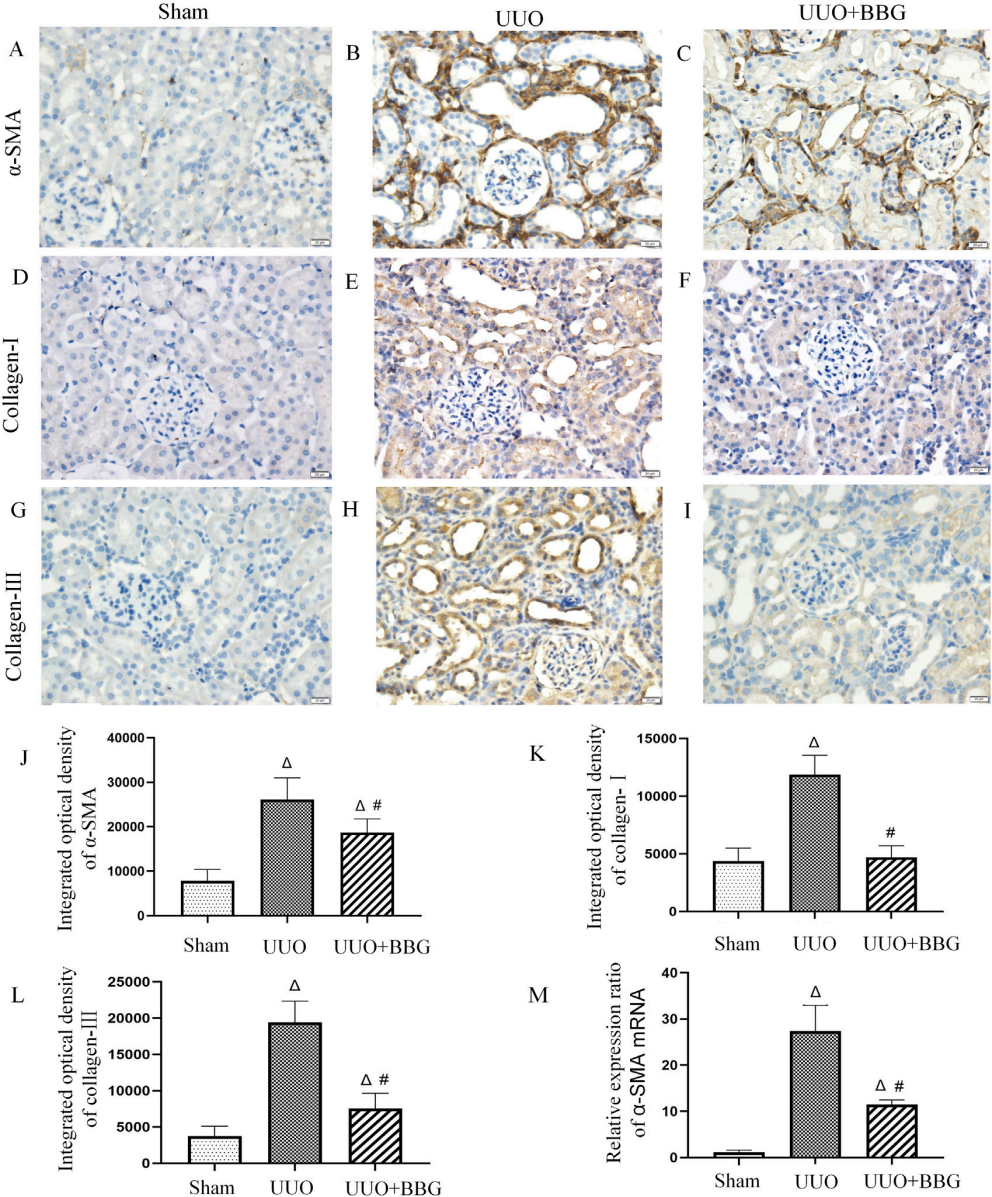
Immunohistochemical staining of α-smooth muscle actin (α-SMA), collagen-Ⅰ and - Ⅲ, and the relative mRNA expression of α-SMA. No positive staining of α-SMA **(A)**, collagen-Ⅰ **(D)**, and collagen- Ⅲ **(G)** is visible in the sham group. The obstructed kidneys show numorous positive staining of α-SMA **(B)**, collagen-Ⅰ **(E)**, and collagen- Ⅲ **(H)**. Compared with the UUO group, BBG treatment reduced positive expression of α-SMA **(C)**, collagen-Ⅰ **(F)**, and collagen- Ⅲ **(I)**. Semi-quantitative analyses of immunohistochemical staining of α-SMA **(J)**, collagen-Ⅰ **(K)**, and collagen- Ⅲ **(L)** show increased protein expression in the UUO group and subsequent reductions after BBG treatment. The notable increase in mRNA expression of α-SMA in the UUO group is reversed via BBG administration **(M)**. Scale bar = 20 μm. Magnification ×400. ∆, P < 0.05 vs. sham group; #, P < 0.05 vs. UUO group.

UUO is a typical model of renal interstitial fibrosis and it has been thoroughly examined by several studies in the past. In the present study, IHC staining and RT-qPCR were performed to clarify whether P2X7R was involved in renal TEMT and interstitial fibrosis. IHC staining demonstrated that the protein expressions of collagen-I and -III had increased in the kidneys of rats after UUO (Figures 3E, H, K, L), compared with the sham group (Figures 3D, G). Significant reductions in the expression levels of collagen-I and -III were noted in UUO + BBG rats (P < 0.05) (Figures 3F, I, K, L). α-SMA functions as a marker of renal intrinsic cell transdifferentiation to myofibroblasts. The expression of α-SMA was not detected in the sham group (Figure 3A). In Figures 3B, J, it is evident that α-SMA protein expression had increased remarkably in obstructed kidneys, in tubular epithelial cells in particular. Similarly, the mRNA levels of α-SMA appeared to increase considerably after UUO (Figure 3M). BBG administration significantly reversed these changes in α-SMA (P < 0.05) (Figures 3C, J, M). Therefore, P2X7R is involved in renal interstitial fibrosis and TEMT.

### 3.4 P2X7R/NLRP3 pathway in renal interstitial fibrosis

**FIGURE 4:**
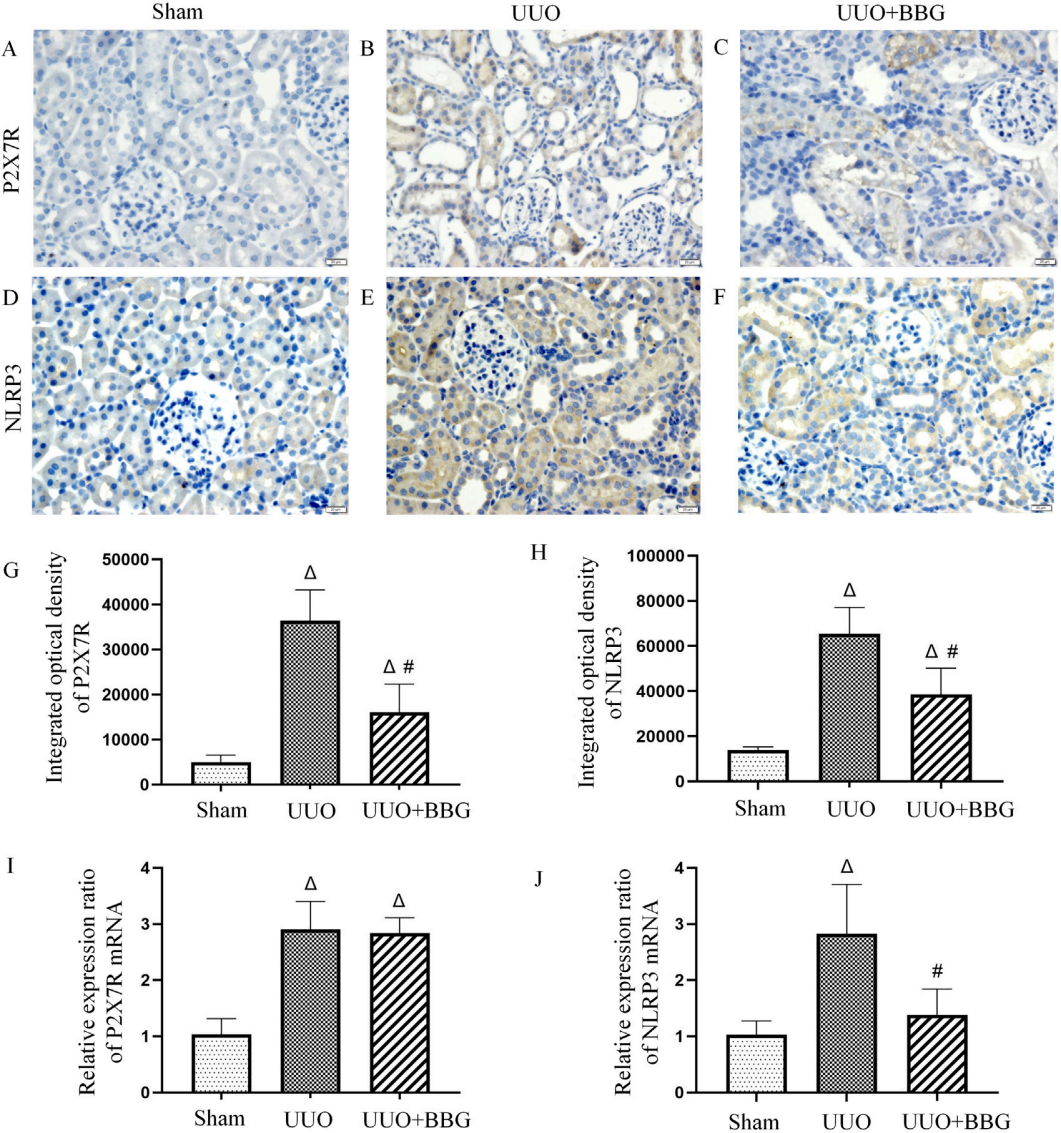
Protein and mRNA expression of P2X7 receptor (P2X7R) and NOD-like receptor protein 3 (NLRP3). No positive staining of P2X7R **(A)** and NLRP3 **(D)** is noted in the sham group. Positive staining of P2X7R **(B)** and NLRP3 **(E)** is significant in the obstructed kidneys. BBG treatment attenuates the protein expression of P2X7R **(C)** and NLRP3 **(F)** after UUO. In semi-quantitative analyses of immunohistochemical staining, an increase in the protein expression of P2X7R **(G)** and NLRP3 **(H)** can be observed in obstructed kidneys and those are significantly reduced following BBG administration. mRNA levels of the P2X7R **(I)** and NLRP3 **(J)** appear to be upregulated in the UUO group. BBG treatment remarkably attenuates the increase in NLRP3 mRNA expression. The mRNA expression of P2X7R did not change in response to BBG treatment. Scale bar = 20 μm. Magnification ×400. ∆, P < 0.05 vs. sham group; #, P < 0.05 vs. UUO group.

Previous studies have shown that the P2X7R/NLRP3 pathway may critically influence in renal injury. To understand whether the P2X7R/NLRP3 pathway contributes to renal interstitial fibrosis, the mRNA and protein expressions of P2X7R and NLRP3 were measured in UUO and BBG-treated UUO rats. The results showed that the positive staining of P2X7R and NLRP3 was more prominent in the UUO group (Figures 4B, E) than in the sham group (Figures 4A, D). After blocking P2X7R with BBG, NLRP3 and P2X7R protein levels appeared to reduce dramatically (P < 0.05) (Figures 4C, F, G, H). In a similar vein, the mRNA expression levels of these two genes were upregulated in obstructed kidneys when compared with control kidneys (Figures 4I, J). BBG treatment ensured that the increase in NLRP3 mRNA expression was adequately attenuated. However, the mRNA expression of P2X7R did not change in response to BBG treatment. These outcomes implied that NLRP3 is downstream of P2X7R in UUO rats and highlighted the important role of the P2X7R/NLRP3 pathway in renal interstitial fibrosis.

### 3.5 Effect of BBG on kidney function

**FIGURE 5:**
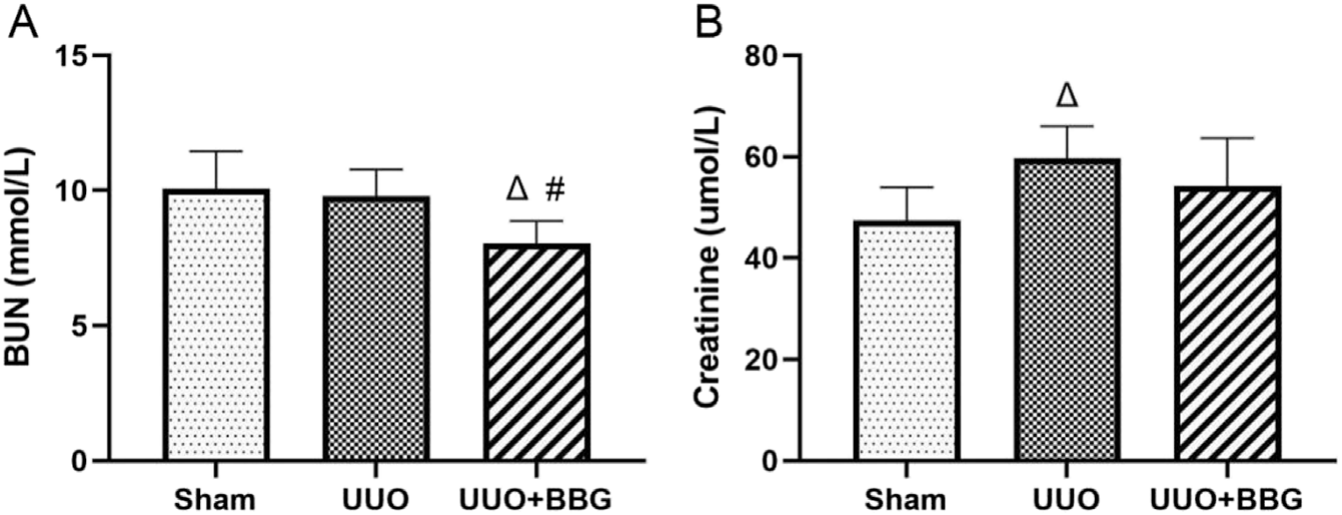
Evaluation of kidney function. Blood urea nitrogen (BUN) concentrations did not increase in UUO rats **(A)**. A reduction in the level of BUN can be observed in the BBG group. BBG administration did not influence the significant increase in serum creatinine in UUO rats **(B)**. ∆, P < 0.05 vs. sham group; #, P < 0.05 vs. UUO group.

Figure 5 shows that serum creatinine concentrations appeared to increase significantly in UUO group when compared with the sham group. BBG treatment was unsuccessful in reducing the serum concentrations of creatinine, albeit these concentrations showed a decreasing tendency (Figure 5B). However, serum BUN concentrations decreased significantly in the BBG group when compared with the UUO group (Figure 5A).

## 4 Discussion

Extracellular ATP release can be triggered by a wide range of stimuli, including mechanical stress, cell damage, inflammation and hypoxia (Solini et al., 2015). Elevated blood pressure-induced ATP release reported increased plasma ATP concentration and induced subsequent immune responses in patients and mice with hypertension (Zhao et al., 2019). In the present study, the ATP concentration in the obstructed kidney showed a remarkable increase. The increase in renal intratubular pressure after UUO was hypothesized to contribute to change in ATP concentrations in the renal tissue. Furthermore, P2X7R was activated. However, the concentration of ATP in kidney tissues did not appear to reduce significantly when UUO rats were treated with BBG; this suggested that blocking P2X7R did not influence ATP release. ATP release-related pathways are complicated and include exocytosis, membrane channel- and transport protein-mediated release, and other mechanisms in some pathophysiological conditions (Dahl, 2015). Connexin 43 and pannexin 1—channel proteins—are reported to mediate ATP release in renal tubular epithelial cells (Sun et al., 2020; Xu et al., 2022). Some studies have speculated that ATP and purinergic receptors are involved in ATP release in astrocytes, erythrocytes and cancer cells (Ohshima et al., 2010; Dahl, 2015). ATP release was controlled by P2X7R using the receptor pore in neuroblastoma cells; this pore acts on P2X7R on the same or adjacent cells to further stimulate ATP release (Gutierrez-Martin et al., 2011). According to our results P2X7R was not involved in the release of ATP in the kidney.

In response to elevated extracellular ATP, P2X7R activated the subsequent signaling pathway. P2X7R’s effect on renal interstitial fibrosis has been examined thoroughly *in vivo* and *in vitro* (Goncalves et al., 2006; Komada et al., 2014; Pereira et al., 2020; Xu et al., 2022). Exposure of Madin-Darby canine kidney cells to the selective P2X7R agonist BzATP induced TEMT to the same extent as exposure to 5 ng/mL TGF-β1 (Zuccarini et al., 2017), a widely accepted profibrotic cytokine. In the present study, collagen deposition and the protein and mRNA expression of P2X7R and α-SMA showed a remarkable increase on day 7 in rats in the UUO group when compared with those in the sham group. This outcome is consistent with a previous study (Goncalves et al., 2006) and indicates the presence of a relationship between P2X7R, renal TEMT, and interstitial fibrosis. In that study, renal interstitial fibrosis was alleviated in P2X7R-knockout mice on days 7 and 14 of UUO; however, P2X7R expression was negative in wild-type mice 14 days after UUO. In the early phase (day 3) of UUO, renal interstitial fibrosis was significantly accompanied by elevated P2X7R expression (Pereira et al., 2020). The effect of P2X7R activation was hypothesized to eventually weaken; however, this claim requires further examination and elucidation. To further evaluate the role of P2X7R in renal fibrosis, IP administration of BBG was performed in UUO rats; this alleviated renal TEMT and interstitial fibrosis. This observation was consistent with two studies in which BBG played a critical role in the attenuation of renal interstitial fibrosis in a salt-sensitive hypertension model and pyelonephritis model (Ji et al., 2012a; Therkildsen et al., 2019). In addition, renal interstitial fibrosis on day 3 after UUO was alleviated via intravenous administration of BBG (Pereira et al., 2020). Additionally, the results of the present study showed that the protein expression of P2X7R was reduced by BBG treatment. Notably, the mRNA level of P2X7R was not affected by the antagonist. P2X7R is formed by 3 homologous subunits, each of which contains two transmembrane-spanning domains that are connected to a large extracellular domain, and these subunits assemble to form a homotrimer. BBG binds an inter-subunit allosteric pocket of P2X7R in a non-competitive manner and inhibits ATP-induced currents (Drill et al., 2021). In the present study, we showed that the antagonism of BBG after binding with the P2X7R protein was not affected by the mRNA expression of P2X7R. However, the specific mechanism requires further elucidation. Although the effects of BBG on renal interstitial fibrosis were clear, renal TEMT and fibrosis-specific markers were higher in rats treated with BBG than in those in the sham group. These results indicated that P2X7R partially contributed to renal TEMT and interstitial fibrosis.

The NLRP3 inflammasome regulates renal inflammation and fibrosis in UUO through interactions with ASC and procaspase-1 to form the inflammasome, which consequently mediates the activation of caspase-1, cleaving cytokine precursors such as interleukin 1β (IL-1β), IL-18, and other cytokine precursors, transforming them into their mature forms and participating in the inflammatory reaction. In addition, activated caspase-1 can mediate the programmed cell death mode known as pyroptosis (Xiong et al., 2021). NLRP3^−/−^ mice exhibited less tubular injury, inflammation, and fibrosis after UUO than wild-type mice (Vilaysane et al., 2010). The outcomes of the present study revealed that NLRP3 expression was upregulated in the UUO rats, which was consistent with previous studies (Ram et al., 2022). Indeed, blocking P2X7R inhibited NLRP3 expression, which consequently indicated that NLRP3 was a downstream molecule of the P2X7 pathway. Contributions of the P2X7R/NLRP3 pathway to renal injury have been confirmed in cases of lupus nephritis. BBG treatment causes a significant reduction in the severity of nephritis and circulating anti-dsDNA antibodies (Zhao et al., 2013). The outcomes observed in angiotensin II-induced hypertension appeared to be relatively similar. The angiotensin II-induced overexpression of the NLRP3 inflammasome was ameliorated via BBG administration (Bautista-Perez et al., 2020).

## 5 Conclusion

Therefore, the P2X7R/NLRP3 pathway—which is influenced by increased extracellular ATP—is involved in renal TEMT and interstitial fibrosis. BBG alleviated renal interstitial fibrosis; however, a notable reduction in serum creatinine concentration was not observed in UUO rats. Some limitations of the present study need to be highlighted. First, the research was performed at a single time point. The long-term effects of the ATP/P2X7R/NLRP3 pathway on renal fibrosis remain unclear. Second, this study only involved the use of an animal model to observe the role of the ATP/P2X7R/NLRP3 pathway in renal interstitial fibrosis. *In vitro* studies are needed to explore the mechanisms underlying these findings. Third, although BBG appeared to have a protective effect on renal TEMT and fibrosis, an in-depth investigation of its pharmacological effects is warranted in further studies.

In conclusion, the findings of the present study provide evidence of the important functions of the ATP/P2X7R/NLRP3 pathway in renal TEMT and interstitial fibrosis. P2X7R antagonists mitigate renal injury and have the potential to be considered effective therapeutic agents for treating renal interstitial fibrosis in the future.

## Data Availability

The data supporting the findings of this study are available from the corresponding author upon reasonable request.
